# Supplementary material for: Conjunctive tuning and cortical geometry shape predictive visual remapping
Source: Res Sq. 2025 Sep 25:rs.3.rs-7536239. Preprint. [Version 1] doi: 10.21203/rs.3.rs-7536239/v1 (PMC12486069; doi:10.21203/rs.3.rs-7536239/v1)
Supplement: 1 [file NIHPPRS7536239V1-supplement-1.pdf]

Figure S1

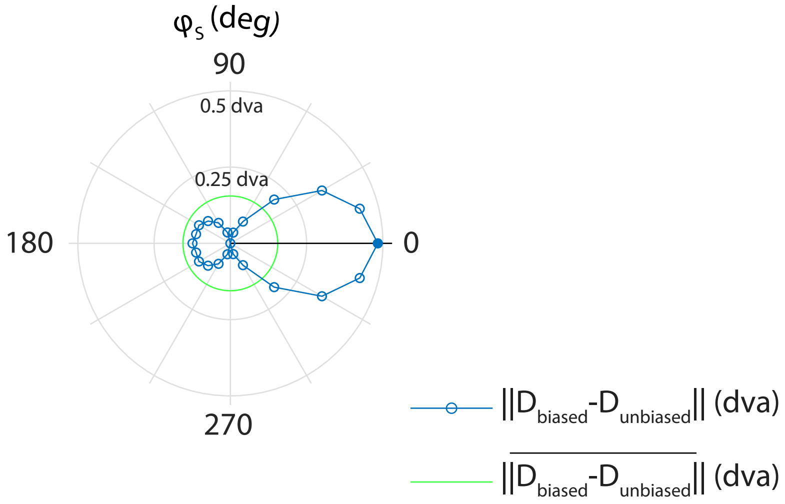

Figure S1. Remapping error induced by bias in the strength of saccade direction selectivity as a function of planned saccade direction.

Black line: direction of SDP along which the bias is introduced by a 40% increase (Fig.1G, left). Green circle: mean error across tested saccadic directions (φ_S). Filled circle: maximum error across φ_S. $PS = 2.8$, $A_{SDP} = 1.68$ for $\varphi_{VF,i} = 0°$ under the biased condition and 1.2 otherwise.

Figure S2

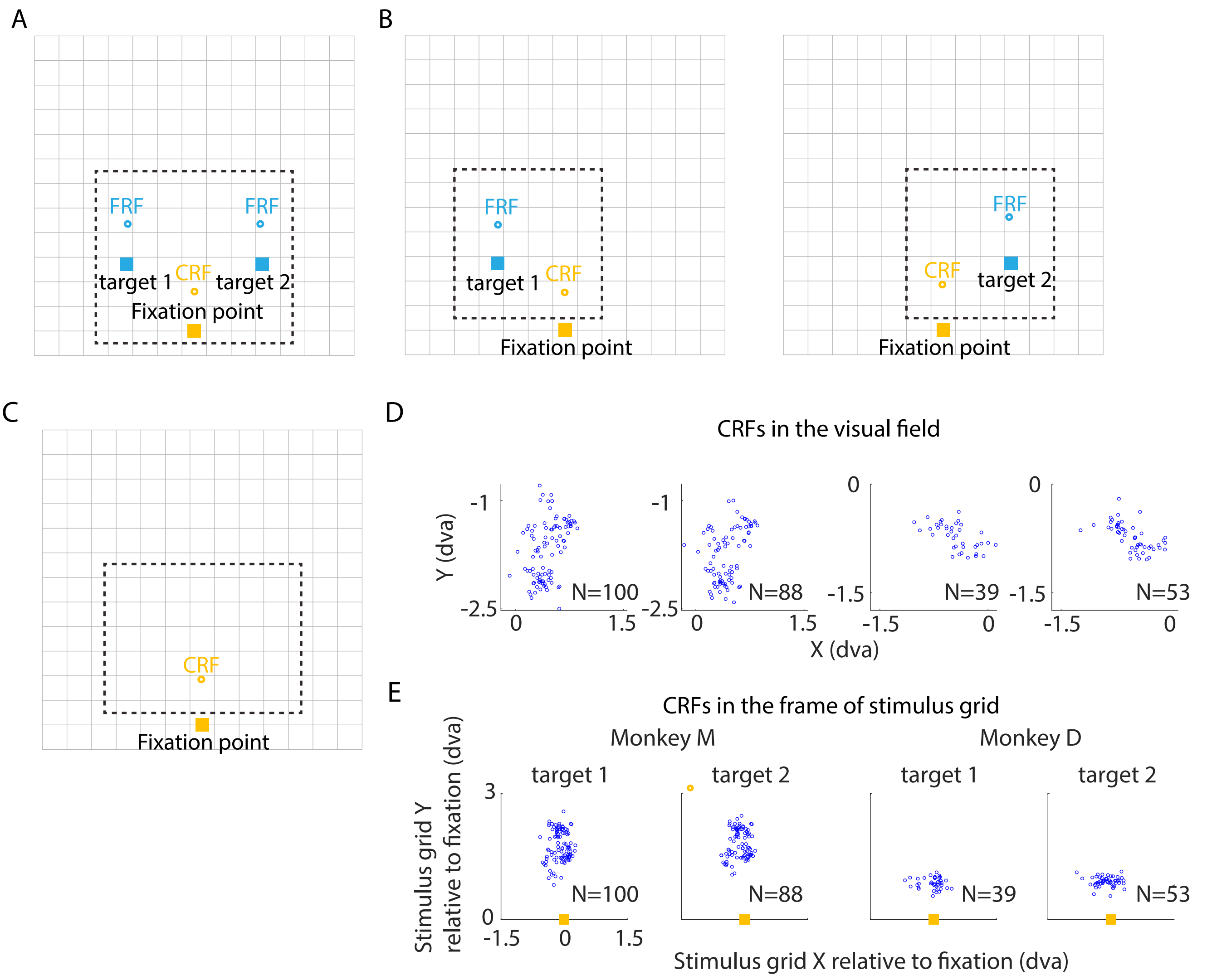

Figure S2. Neuronal responses and RFs under a cued saccade task

(**A**) The area of stimulus locations used to compute the responses under various saccade directions. (**B**) Visual regions of interest for RF searching during saccade planning for various saccade directions. (**C**) Visual region of interest for RF searching during the pre-planning period. (**D**) Distribution of current RFs (CRFs) for recorded neurons exhibiting forward remapping under various saccade directions for each monkey. (**E**) Same as (D) but shown in the reference frame of the stimulus grid, where the coordinates of fixation point and saccade targets were fixed across sessions.

Figure S3

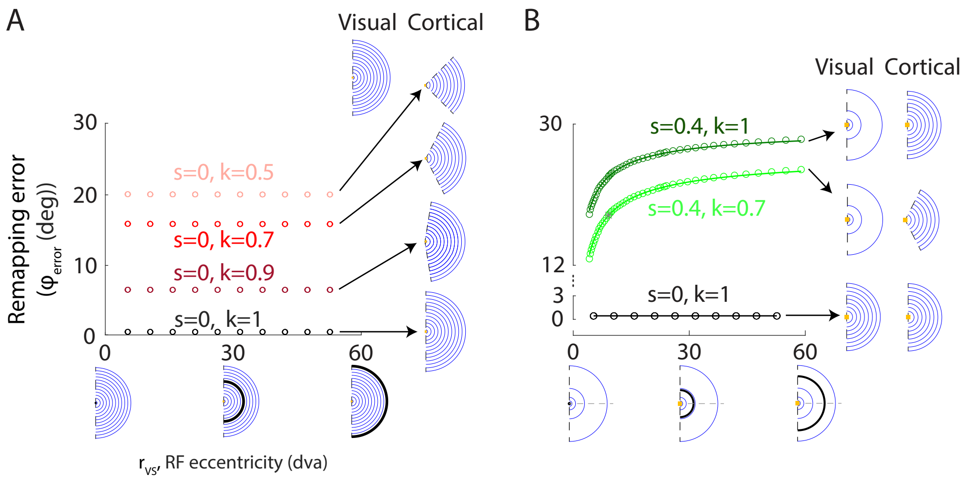

Figure S3. Remapping error under polar angle contraction with eccentricity-independent cortical magnification

(A) Same as Fig. 3D but under conditions of eccentricity-independent cortical magnification (CM) ($s = 0$) across various extents of polar angle contraction (k). The remapping error is independent of CRF eccentricity. (B) Light green: same as Fig. 3D but under conditions of both eccentricity-dependent CM ($s = 0.4$) and polar angle contraction ($k = 0.7$). Black: uniform transformation as shown in Fig. 3D. Dark green: eccentricity-dependent cortical magnification (CM) only as shown in Fig. 3D. Grey asterisk: transformations considered in Fig. 3B, C.

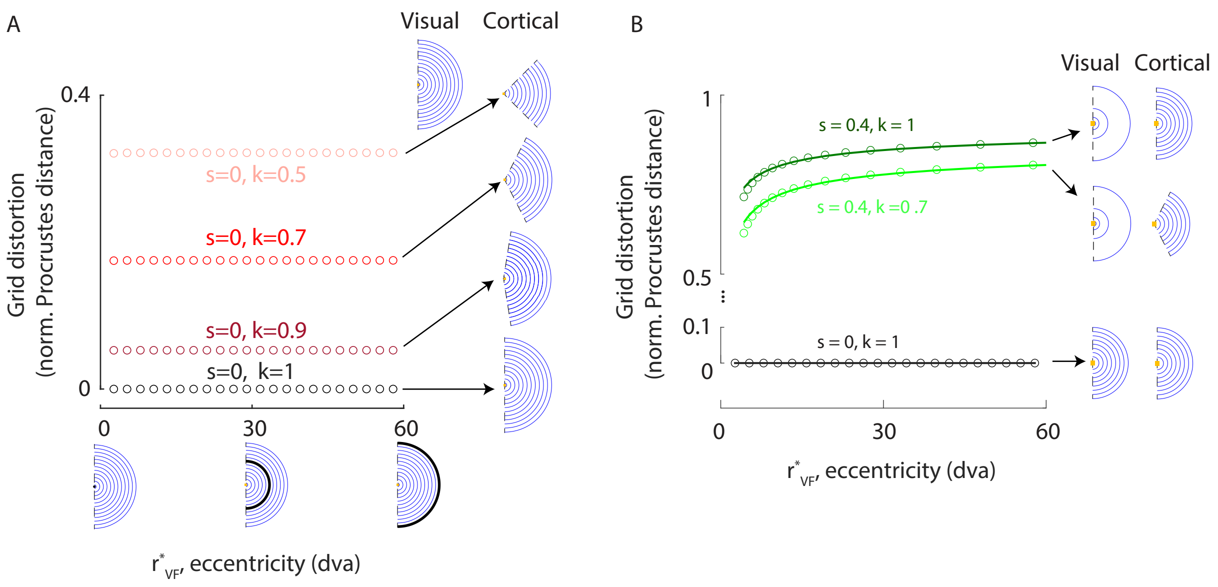

Figure S4. Grid distortion under polar angle contraction with eccentricity-independent cortical magnification

(A) Same as Fig. 5B but under conditions of eccentricity-independent CM ($s = 0$) across various extents of polar angle contraction (k). The grid distortion is independent of the eccentricity of network location. (B) Light green: same as Fig. 5B but under conditions of both eccentricity-dependent CM ($\mathrm{s} = 0.4$) and polar angle contraction ($\mathrm{k} = 0.7$). Black: uniform transformation as shown in Fig. 5B. Dark green: eccentricity-dependent cortical magnification (CM) only as shown in Fig. 5B.

Figure S5

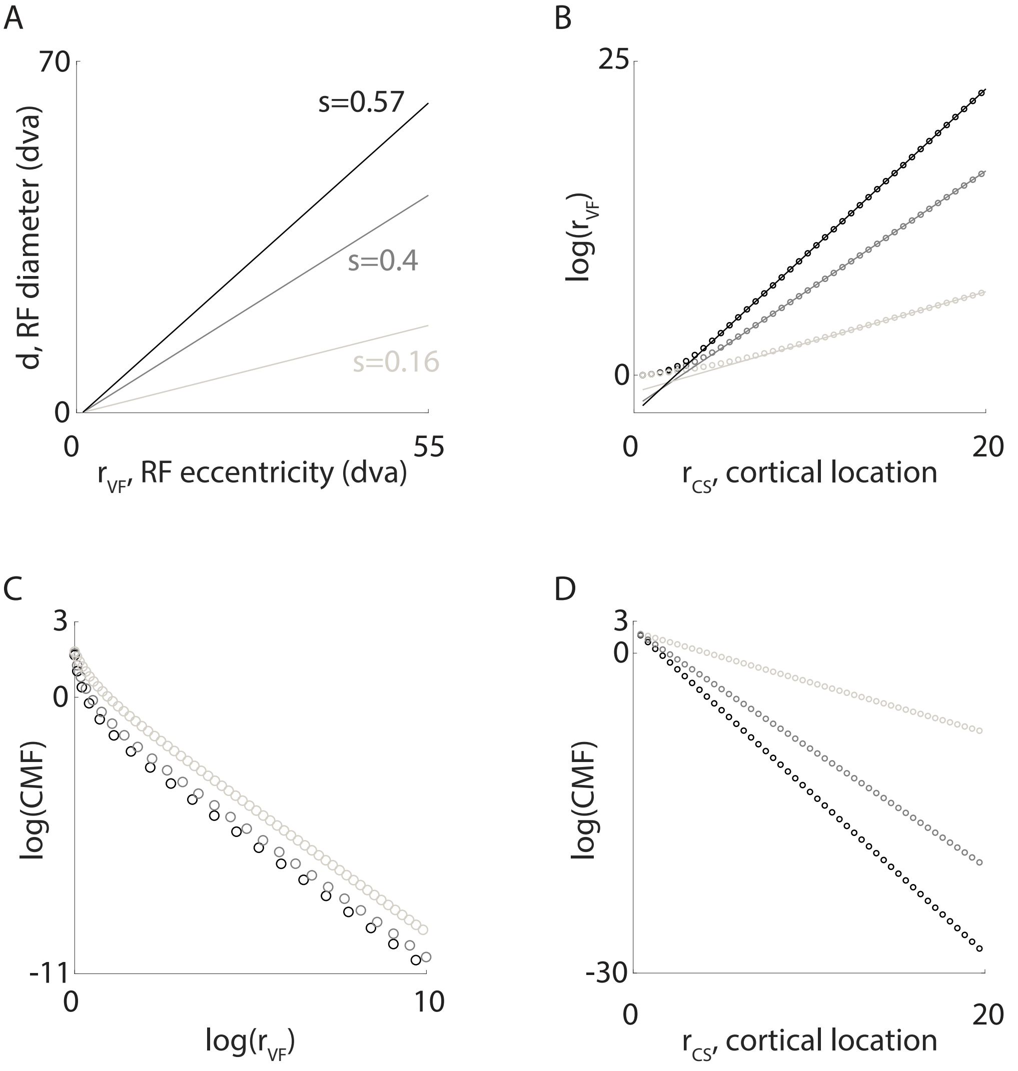

Figure S5. Constructing eccentricity-dependent cortical magnification

(**A**) Linear relationships between RF diameter (d) and RF eccentricity (r_{VF}) explored in the study. (**B**) Corresponding $log(r_{VF})$ as a function of cortical location (r_{CS}), constructed using the linear relationship shown in (A). Circles: constructed data. Lines: theoretical linear relationships (Eq. 13-14). (**C**) Corresponding CM factor (CMF), approximated by forward difference methods based on (**B**). (**D**) Same as (C) but as a function of r_{CS}. $D_c = 0.5$, $d_1 = 0.1$, $\mathrm{r}_{\mathrm{VF},1} = 1$.
